# Interface-edited solid-state NMR to study cell interfaces

**DOI:** 10.1038/s42004-025-01473-7

**Published:** 2025-03-22

**Authors:** Thomas Kress, Melinda J. Duer

**Affiliations:** Yusuf Hamied Department of Chemistry, University of Cambridge, Cambridge, UK

**Keywords:** Solid-state NMR, Glycobiology, Biomaterials - cells

## Abstract

Cell membrane interfaces, including the glycocalyx, play a crucial role in regulating signaling and molecular interactions, yet their molecular composition remains challenging to study in intact cells. Existing techniques often require extensive sample preparation or lack specificity for probing interfacial components directly. Here, we introduce a solid-state nuclear magnetic resonance (ssNMR) tool to fingerprint the molecular structure of the cell glycocalyx in intact cells within their native environment, offering insights relevant to drug delivery, tissue engineering, and biomedical research. Building on Goldman-Shen cross-polarization (CP) experiments, which exploit proton spin diffusion to generate ^13^C spectra near cell membranes, our enhanced approach provides spectral information from the membrane interface and its surroundings, probing a region up to 10 nm. Using interface-edited CP (1D) and PDSD (2D) spectra, we demonstrate spectral fingerprints of the mammalian cell glycocalyx. This method opens new avenues for studying cell interfaces in a dehydrated yet native-like state, preserving membrane composition and advancing structural biology.

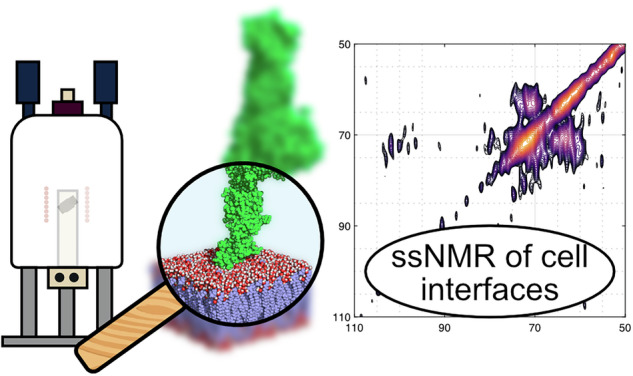

## Abstract (web summary)

Cell membrane interfaces, including the glycocalyx, play a crucial role in regulating signaling processes, yet its molecular composition remains poorly understood due to a lack of tools for probing interfacial components in intact cells. Here, the authors present a solid-state nuclear magnetic resonance approach that selectively probes the glycocalyx and its surroundings, providing molecular-level insights into cell interfaces in their native extracellular environment.

## Introduction

The interfacial region proximal to biological cell membranes holds immense significance due to its crucial role in regulating the interactions between cells and their environment^1^. In particular, the glycocalyx formed by sugar-modified proteins at the cell plasma membrane is an important aspect in controlling cell signaling through their influence on the complex, dynamic interactions between cell membrane proteins and their ligands^1^. However, there is relatively little known about how the glycocalyx is involved in cell signaling because of the difficulty of studying the composition of the glycans involved. Current methods of studying the glycocalyx involve imaging and staining approaches are also commonly used to visualize the glycocalyx and study its structure and distribution^2^, or the disruption of the cell membrane, degradation of the glycoproteins, and subsequent analysis of the sugar components through e.g., LC-MS^3^. There is the need for in-situ methods that preserve cellular organization while enabling molecular fingerprinting of glycan components to study their structure and composition. Such approaches would facilitate direct comparisons between cell types or environments without the need for destructive sample preparation.

Membrane interfaces, i.e., regions separating different cell environments, are intrinsically dilute components within a cell-matrix system and even within cells. Conventional NMR spectroscopy monitors signals from the bulk of the sample with little discrimination in favor of the interface. This limitation has traditionally confined NMR applications to simpler model systems, such as purified proteins embedded in membrane mimetics. However, advancing the ability of NMR to tackle complex biological systems would open opportunities to study interfacial regions in their near-native state. Such advances must overcome the challenge of separating weak, broad spectral signals from interfacial regions amidst stronger signals from bulk cellular contents.

However, NMR spectroscopy offers the potential for carefully designed experiments that can edit spectra to isolate signals of interest. One approach that has proven effective is targeted Dynamic Nuclear Polarization (DNP)^4^ and surface-enhanced DNP^5^ which enhance NMR signals by colocalizing the polarizing agent with the region of interest. This strategic placement of the polarizing agent has been achieved using molecules with an affinity to cellular components^6–10^, or directly attached to proteins using covalent bonds^11–13^, which allowed for the selective amplification of weak NMR signals from these specific regions, overcoming the masking effects of stronger signals from bulk cellular content. The need for spatially-localized DNP radicals places the further requirements of verifying the DNP radical location and its non-interference with cell membrane structure. These challenges, combined with the need for specialized equipment, make selective DNP less practical for routine or comparative studies of multiple samples. Simplified approaches that provide sufficient spectral selectivity without the use of enhancement agents would greatly benefit the field.

**Fig. 1:**
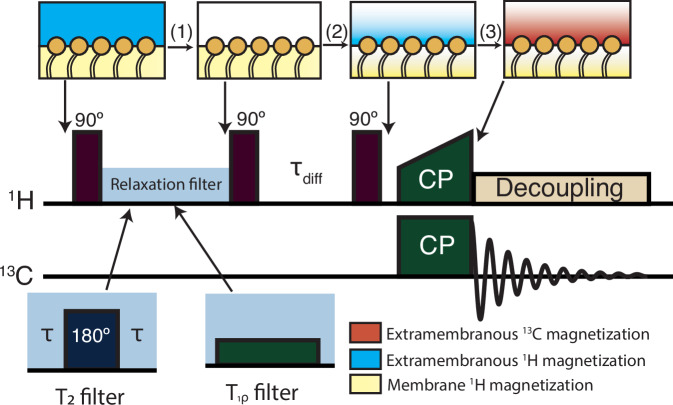
A Goldman-Shen experiment can be divided into three steps for the investigation of cell glycocalyx. i) ^1^H magnetization from the lipid cell membrane is selected, (ii) ^1^H magnetization diffuses to the extramembranous region, (iii) cross-polarization from ^1^H to ^13^C for detection of an interface-edited ^13^C spectrum.

Goldman-Shen ssNMR experiments^14^ use relaxation filters to discriminate signals from parts of the sample with different molecular mobilities (Fig. 1). They can be divided into three parts (i) the selection of typically ^1^H NMR signals associated with contrasting relaxation properties, (ii) a spin diffusion time that transports the ^1^H magnetization to regions with lower net magnetization, (iii) the detection of NMR signals of interest. Many variations of Goldman-Shen experiments have been reported using different types of relaxation filters, including T_2_^⋆^^14^, T_2_^15^^15^, WAHUHA (T_1ρ_)^16^, or T_1_^16^. In some cases, these experiment has been combined with cross-polarization step prior to detection to enhance the spectral resolution and extend the range of observable nuclei, including dilute ones like ^13^C, by transferring polarization from selected ^1^H signals^16^.

Previous studies have primarily investigated protein-water interactions using fully ^13^C-^15^N enriched proteins expressed in *E. coli* and reconstituted in lipid membranes to create model systems^15,17^. While these approaches have yielded valuable insights into membrane protein dynamics in controlled environments, our work takes a fundamentally different direction. We focus on the proximity of cellular components to lipid membranes in intact cellular systems, rather than in purified or model-based samples.

Phospholipids in cell membranes, with their characteristic mobility on the micro- to nanosecond timescale, present an ideal target for spatial editing using Goldman-Shen NMR methods. Unlike model systems, our samples preserve the complexity of native cellular mixtures, resulting in broad spectral features that demand advanced spectroscopic techniques for interpretation. This enables us to study membrane-associated phenomena in a more biologically relevant context, even with the limitations imposed by dehydrated samples.

Addressing the complexity of intact cellular systems—where interfacial components coexist with a multitude of other cellular elements—requires methodologies that allow selective detection of interfacial signals while preserving the spatial arrangement of cellular components to maintain biological context. Advances in ssNMR are uniquely positioned to meet these challenges, offering powerful tools to study interfacial phenomena with minimal disruption to the native-like organization of samples.

Goldman-Shen experiments were first used to measure domain sizes in semicrystalline polymers^18,19^, using the ^1^H magnetization selected from mobile, amorphous polymer components. Then, Kumashiro et al. used water ^1^H magnetization to study the spatial localization and orientation of membrane proteins incorporated within lipid vesicles^15^; this allowed the determination of which parts of a membrane protein were exposed to water, and the insertion depth of membrane protein segments into phospholipid membranes. Later, Huster et al. used the reversed experiment^20^, where the proton magnetization stored within a phospholipid bilayer was used to study the insertion depths of membrane proteins using the build-up curves of protein signals.

One advantage of the Goldman-Shen approach to spectral editing is that it can also in principle select signals according to the distance from the initial ^1^H source. This is potentially invaluable for NMR fingerprinting of the cell glycocalyx, as spectra can potentially be recorded in which the glycan signals have intensities that depend on both their abundance *and* their distance from the cell membrane. This increases the information content of the spectra over a single bulk average NMR spectrum of the glycocalyx, even if recording such a spectrum was possible.

We here propose a new version of the Goldman-Shen experiment to achieve interface-editing with ssNMR and record ssNMR spectra of the crucial interface regions beyond the cell membrane. As in Huster et al.^20^, the GSCP experiment relies on the different molecular mobilities of cell membrane phospholipids compared to molecular mobility in the rest of the sample to select ^1^H magnetization from the cell membrane via its ^1^H magnetization relaxation properties. Here we show that ^1^H rotating frame relaxation can be used as an efficient filter for spectral editing to study the interfacial regions around cell membranes. Once the ^1^H magnetization of the cell membrane is selected via the relaxation filter, a gradient of ^1^H magnetization occurs at the cell interface, which drives ^1^H spin diffusion into the interface region; cross-polarization of the ^1^H magnetization to ^13^C nuclei then yields a high-resolution ssNMR spectrum of the cell interface (Fig. 1).

## Results and discussion

### Selection of lipid ^1^H magnetization

Phospholipid ^1^H can be expected to exhibit much longer T_2_ and T_1ρ_ relaxation times than any other components found in tissue samples, and thus both T_2_ and T_1ρ_-based filters could in principle be used to select phospholipid ^1^H magnetization. We first addressed whether a ^1^H T_2_ or T_1ρ_ filter results in a better selection of cell membrane phospholipid ^1^H magnetization. We use as our test sample ^13^C glucose primary bovine vascular smooth muscle cells (VSMCs) grown in vitro for only a short time period beyond the cells reaching confluence, in order to maximize the proportion of the sample which cellular interface with their surrounding extracellular matrix. The samples were freeze-dried to remove mobile (bulk) and unbound water, as the overwhelming signal intensity of water make the selection of lipid magnetization incompatible with hydrated samples. This approach contrasts with that of others, who transferred water magnetization to membrane proteins^17^, and thus used a fully hydrated sample. Retaining bulk water dramatically reduces membrane ^1^H signal selectivity and furthermore, occupies sample space within the NMR rotor, thereby lowering the signal-to-noise ratio. Because no cryoprotectants were used to protect the cells during the freezing step, it is likely that large-scale structures, such as the plasma membrane, rupture during the freezing step, significantly reducing overall cell viability. However, on a local level, portions of the cell membrane appear to remain intact. This is supported by the sharp lipid signals observed in the ^13^C INEPT spectrum (Fig. S1), which are characteristic of well-organized lipid regions. Cryoprotectants, while effective in preserving large-scale membrane integrity by forming a glassy matrix, often introduce molecular mobility that leads to prolonged T2 and T1ρ relaxation times. Such conditions are not compatible with our approach, which relies on relaxation filtering for spectral editing.

**Fig. 2:**
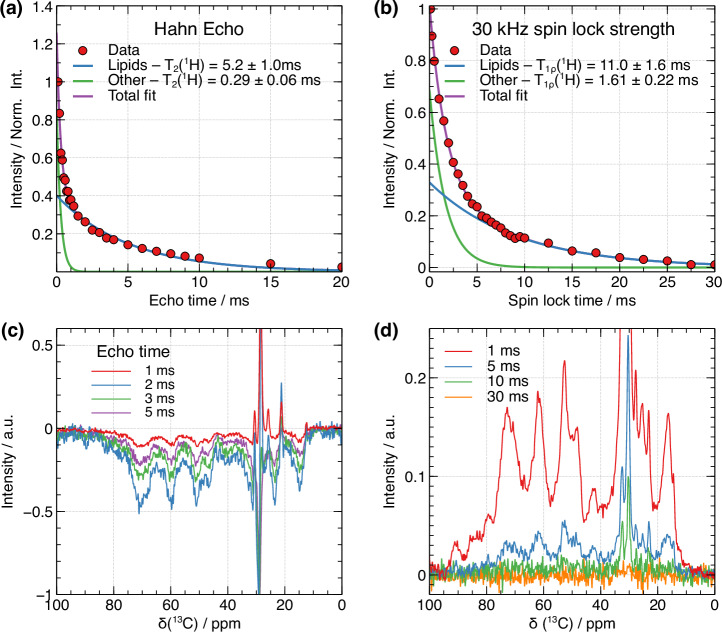
Comparison of Hahn echo filters vs spin-lock filters. **a** Measurement of T_2_(^1^H) relaxation times of phospholipids and slower-relaxing components in an in vitro U-^13^C glucose labeled tissue sample (cells plus cell-derived extracellular matrix) grown from VSMCs with a spin echo experiment; **b** Measurement of T_1ρ_(^1^H) relaxation times of phospholipid and slower-relaxing ^1^H signals under a 30 kHz spin-lock. ^1^H magnetization decay curves were fitted with a bi-exponential function accounting for the phospholipid, and slower-relaxing components. **c** ^13^C Goldman-Shen CP spectra recorded using a spin echo relaxation filter for the specified echo times to select lipid proton magnetization (**d**) ^13^C Goldman-Shen spectra using a T_1ρ_(^1^H) relaxation filter for the specified lengths of time to select lipid ^1^H magnetization.

The ^1^H spectra of VSMC samples at 10 kHz MAS (Fig. S2A) features the superposition of sharp signals, onto a broad background signal arising from other components in the sample with much shorter T_2_(^1^H). The sharp signals originate from highly mobile lipid components which are linked to a single network of coupled protons, as evidenced by TOCSY ^1^H-^1^H spectra that only retains the signal from the mobile molecular components owing to the long spin-lock pulse used (Figure S2B). In general, a spin echo delay of >5 T_2_ can be expected to effectively suppress the transverse magnetization, so an effective T_2_(^1^H) filter to remove protein ^1^H transverse magnetization whilst retaining phospholipid transverse magnetization would require the phospholipid T_2_(^1^H) to be significantly longer than 5 times the protein T_2_(^1^H). For our VSMC samples, we fitted T_2_(^1^H) values for the lipid (acyl chain) (5.2 ± 1 ms) and slower-relaxing (0.29 ± 0.06 ms) ^1^H signals (Fig. 1a). This tenfold difference between lipid and slow component T_2_(^1^H) could be used to select lipid ^1^H magnetization, as a 2 ms echo time would be expected to effectively remove the protein ^1^H magnetization whilst retaining ca. 30% of the initial lipid ^1^H magnetization. However, we found in practice that the selection of proton magnetization based on T_2_(^1^H)-based relaxation filters led to artefacts in the resulting GSCP spectra (Fig. 2c), most likely because of T_1_(^1^H) relaxation effects and ^1^H spin diffusion occurring during the spin echo.

Instead, we trialed a T_1ρ_(^1^H) filter and found that it was equally able to select lipid magnetization (Fig. 2b), and resulted in a cleaner lipid ^1^H signal selection in our hands (Fig. 2d and Fig. S3) due to reduced T_1_(^1^H) relaxation during the spin-lock. Moreover, the T_1ρ_ filter was found to be more versatile than a T_2_(^1^H) filter because it avoids the need for the rotor synchronization necessary in the spin echo experiment.

**Fig. 3:**
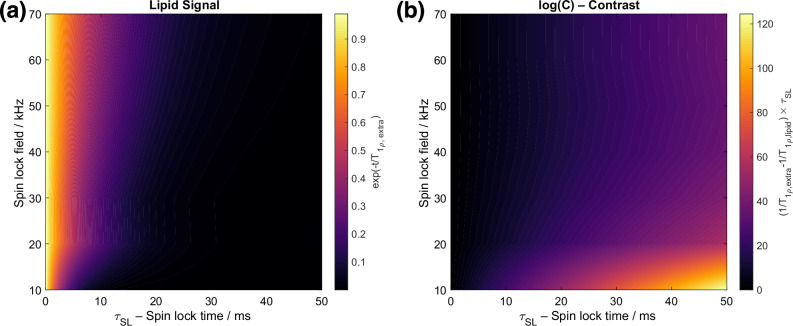
Optimization of spin lock field strength and duration for GSCP experiments. **a** Variation of the phospholipid ^1^H signal intensity after the T_1ρ_(^1^H) filter as a function of spin-lock pulse amplitude and (y-axis) and length (x-axis); **b** Variation of the ^1^H magnetization relaxation contrast defined in Eq. (1). In both cases, the plotted data is obtained from experimental T_1ρ_ (^1^H) relaxation data on an in vitro tissue sample of VSMCs plus the extracellular matrix they deposit (see Experimental methods for details of the sample).

To optimize the *T*_1ρ_(^1^H) filter to achieve (i) best selectivity of the lipid magnetization over the other components, and (ii) best sensitivity for interface-edited experiments, we performed *T*_1ρ_(^1^H) measurements for spin lock field strengths ranging from 10 to 80 kHz and performed a bi-exponential fit on each experiment to obtain the relaxation time constants for lipid and extramembraneous ^1^H signal components respectively. Figure 3a shows how much lipid ^1^H signal remains after the relaxation filter at each spin lock field strength, and Fig. 3b shows the relaxation contrast, defined here as the ratio between the lipid ^1^H signal intensity versus remaining extramembraneous ^1^H signal intensity:

1 $$C\,=\,\frac{{M}_{{{\rm{lipid}}}}\times {\mathrm{exp}}(-{\tau }_{{SL}}/{T}_{1\rho ,{{\rm{lipid}}}})}{{M}_{{{\rm{extra}}}}\,\times {\mathrm{exp}}(-{\tau }_{{SL}}/{T}_{1\rho ,{{\rm{extra}}}})}$$

where ${T}_{1\rho ,{extra}}$ and ${T}_{1\rho ,{lipid}}$ are the ${{{\rm{T}}}}_{1{{\rm{\rho }}}}({\scriptstyle{1}\atop} \!{{\rm{H}}})$ relaxation times of the extramembranous and lipid ^1^H signal components respectively, and ${\tau }_{{SL}}$ is the spin lock time. We find that the choice of spin-lock parameters should balance between retaining phospholipid magnetization and achieving effective relaxation contrast (Figs. 3 and S4). A stronger spin-lock field prolongs ${T}_{1\rho }$ relaxation times by more efficiently “locking” transverse spins along the pulse axis. However, the dipolar interactions in rigid, extramembranous components are stronger, requiring a more intense spin-lock field to effectively spin-lock their magnetization. In contrast, the lipid magnetization, influenced by residual molecular motion, is more easily spin-locked. Thus, for selecting lipid magnetization, weaker and longer spin-lock pulses are more effective, providing better relaxation contrast without overly suppressing the lipid signals. We typically used intermediate spin-lock conditions (30 kHz spin-lock pulse amplitude and 15 ms spin-lock pulse length) to satisfy the need for both relaxation selectivity and spectral sensitivity.

### Spatial selectivity and diffusion of ^1^H magnetization from the lipid cell membrane to extramembranous regions

Having selected the required membrane ^1^H magnetization, the Goldman-Shen CP pulse sequence then allows the selected ^1^H magnetization to diffuse from the membrane to the interfacial regions of interest. We next needed to understand to what distance from the membrane the selected membrane ^1^H magnetization is likely to diffuse to for a given spin diffusion time, in order to be able to interpret interface-edited spectra in terms of the molecular species they will highlight. Thus we next modeled the ^1^H spin diffusion process from membrane to extramembraneous regions using heat equations^21^:

2 $$\frac{\partial m\left({{\boldsymbol{r}}},t\right)}{\partial t}=\nabla \cdot \left[D\left({{\boldsymbol{r}}}\right)\nabla m\left({{\boldsymbol{r}}},t\right)\right]$$

Where $m\left({{\boldsymbol{r}}},t\right)$ is the nuclear magnetization for the spin of interest depending on the position in the system ${{\boldsymbol{r}}}$ to account for the system heterogeneity, $D\left({{\boldsymbol{r}}}\right)$ is the diffusion coefficient which depends on the strength of ^1^H-^1^H dipolar interactions, and $\nabla$ is the gradient operator.

We used Eq. (2) to perform numerical simulations of 1D spin diffusion in a model consisting of a “hot” lipid domain initially containing all the ^1^H magnetization at time 0, with an internal ^1^H spin diffusion coefficient D_lipid_, which is put in contact with a “cold” extramembranous domain with a ^1^H spin diffusion coefficient D_extra_. We measured the proton spin diffusion coefficient in the extramembranous space D_extra_ to be 0.28 nm^2^.ms^-1^ using hole-burning sequences developed by Chen et al. (Figs. S5–6)^22,23^. This value is comparable to the 0.33 nm²/ms measured for polystyrene at the same MAS rate, as well as to values reported in the literature^22^. This similarity is expected because spin diffusion is driven by proton-proton dipolar interactions, and amorphous organic solids typically exhibit similar proton densities and molecular motion, leading to a comparable dipolarly-coupled network of protons and thus similar diffusion coefficients^22^.

**Fig. 4:**
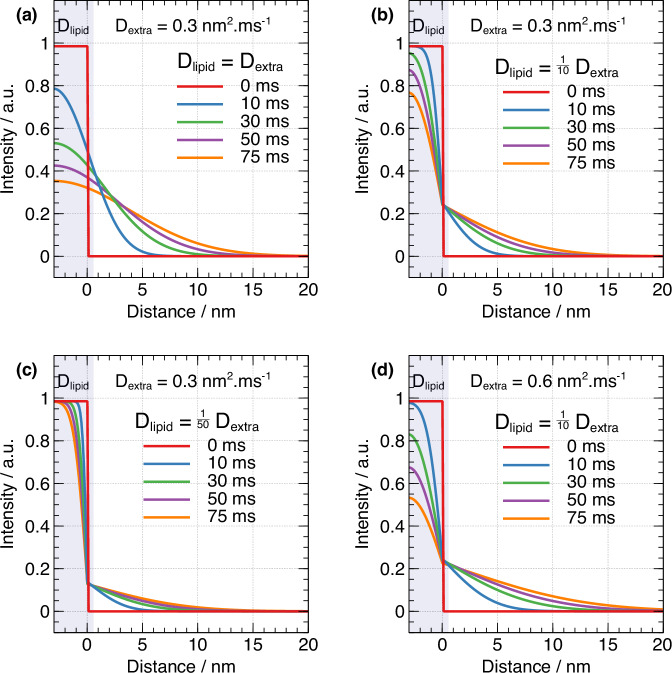
Simulations of 1D ^1^H spin diffusion in a system containing two domains (“lipid” and “extra”) with different intrinsic spin diffusion coefficients as a function of spin diffusion time (x-axis). The ^1^H magnetization is initially stored uniformly across the “lipid” domain (left hand side in each plot, gray shading) of depth 3 nm. **a**–**d** plot the ^1^H magnetization intensity (y-axis) as a function of distance from the “lipid” domain interface with the “extra” domain for different spin-diffusion times (shown as different colored lines on each plot). Each plot is for a different set of D_lipid_ and D_extra_ values.

Figure 4a illustrates simulations of the magnetization profile in our system, where the magnetization is initially stored in the lipid membrane domain, assuming a uniform spin diffusion coefficient across the sample (D_lipid_ = D_extra_). In this scenario, the magnetization quickly spreads away from the cell membrane. However, the averaging of homonuclear ^1^H dipolar interactions within the membrane, caused by phospholipid molecular motion, reduces the effective spin diffusion coefficient in the cell membrane compared to the extramembranous domain (D_lipid_ ≤ D_extra_). This reduction in dipolar coupling, combined with the limitations of hole-burning experiments for accurately determining D_lipid_, required us to assume a constant lipid spin diffusion coefficient in our simulations.

Our results show that a lower D_lipid_ relative to D_extra_ introduces a discontinuity in the magnetization profile near the interface (Fig. 4b–d). The magnetization at the interface remains nearly constant over time, indicating a steady-state regime where the membrane acts as a reservoir of magnetization that is not rapidly depleted. Comparisons between different lipid diffusion coefficients (Fig. 4a, b) highlight that overall magnetization flow is limited by diffusion within the lipid membrane, which serves as the primary reservoir. Once magnetization reaches the interface, it rapidly flows into the extramembranous space.

Therefore, D_lipid_ primarily impacts the magnitude of the steady-state magnetization at the interface, determining the total magnetization transferred to the outer domain, without altering the spatial distribution of magnetization in the extramembranous region. In contrast, D_extra_ dictates the extent of magnetization penetration into the extramembranous space. This suggests that while D_lipid_ affects the signal-to-noise ratio of interface-edited spectra, it has minimal influence on the extramembranous magnetization profile.

**Fig. 5:**
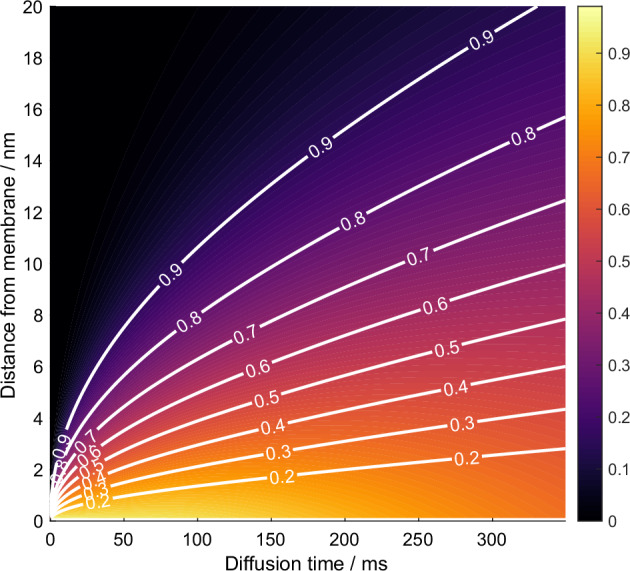
Calculated spatial distribution of ^1^H magnetization in the extramembrane region as a function of spin diffusion time. The simulation was based on the 1D spin-diffusion model described in text and the conditions of Fig. 4b, i.e., D_extra_ = 0.3 nm^2^·ms^-1^ (Fig. S5 for measurement). Distances (y-axis) are reported from the edge of the cell membrane, i.e., close to the separation between fatty acid tails and the lipid headgroup. The white contour lines indicate the levels of the cumulative distribution function integrated over the distance axis. The contour labeled 0.9 indicates the boundary below which 90% of the magnetization is contained, characterizing the spatial spread of the magnetization relative to the cell membrane.

Figure 5 gives a contour plot of the extramembraneous ^1^H magnetization intensity as a function of the spin diffusion time and distance from the membrane-extramembrane interface, which we take here as the phospholipid acyl chain-head group junction, on the basis that it is acyl chain ^1^H magnetization that is selected by the relaxation filter in the GSCP experiment. The white contour levels, which were empirically found to be proportional to $\sqrt{{D}_{{\mbox{extra}}}{\tau }_{{\mbox{diff}}}}$ show how the ^1^H magnetization is spatially distributed within the extramembranous region and describe the ^1^H spin diffusion lengthscale. We estimate that ca. 80% of the initial ^1^H magnetization can be found within 4.5 nm of the source at 50 ms spin diffusion time. We will use this plot hereafter to characterize the lengthscale of proton spin diffusion in experimental examples. Comparing the distances over which the selected ^1^H magnetization can diffuse with the lengthscales of membrane structures, it is clear that we can expect to obtain GSCP ^13^C signals from well beyond the phospholipid headgroups, including from the larger membrane proteins such as integrins that bind cells to the extracellular matrix, and glycosylation sugars.

### Validating distance scales in GSCP experiments: evidence of magnetization transfer beyond the cell membrane

**Fig. 6:**
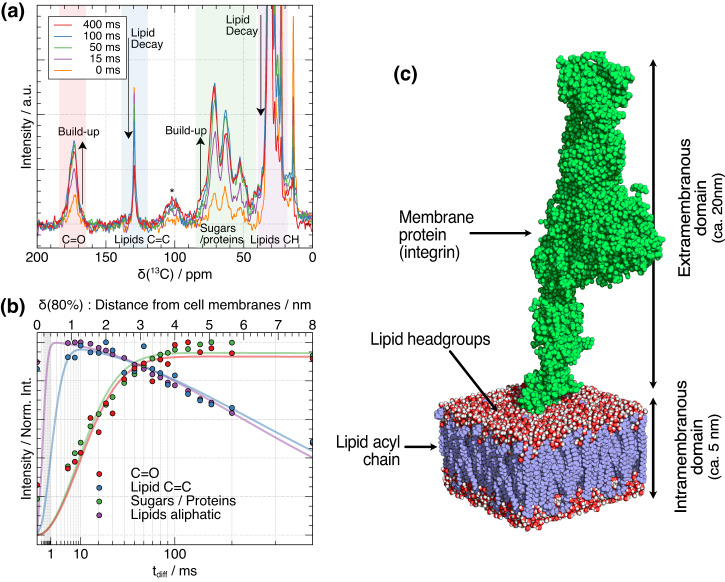
Spin diffusion dynamics in GSCP experiments, highlighting how ^1^H magnetization propagates from the lipid membrane into the surrounding environment over time. **a** Selected GSCP experiments recorded with different spin diffusion times on VSMC samples grown with U-^13^C-glucose. The spectra show changes in relative ^13^C intensity as ^1^H magnetization is transferred out of cell membrane through spin diffusion. In particular, lipid membrane signals decay whereas other signals build up, and before decaying at long spin diffusion times due to ^1^H spin-lattice relaxation. * labels the position of a carbonyl spinning sideband (**b**) Plot of the build-up and decay of the integrated ^13^C signal intensity of lipids and sugars with increasing spin diffusion time. A biexponential fit is displayed to help with the visualization: $I({t}_{{diff}})={I}_{0}\,\exp (-{t}_{{diff}}/{T}_{{BU}})\times \,(1-\exp (-{t}_{{diff}}/{T}_{1}))$ (**c**) Length scale of cell membranes and membrane proteins (integrin, pdb code 2vdo). Proton magnetization initially stored in the lipid membrane diffuses through lipid headgroups to the extramembranous domain. Lipid measurements adapted from Nagle et al.^31^.

Figure 6a shows selected GSCP experiments recorded with different spin diffusion times on VSMC samples grown with U-^13^C-glucose. This ^13^C-source is expected to ^13^C label protein glycosylation sugars, including the glycocalyx that surrounds the cell and the glycerol moieties in the phospholipid headgroups^24^ as well as the ribose/ deoxyribose rings of RNA, poly(ADP ribose)/DNA and some non-essential amino acids, e.g., Gly, Ser, Ala. The integrated intensity of the peaks highlighted with colored boxes is plotted against spin diffusion time in Fig. 6b. For short spin diffusion times (<100 ms), the NMR signals of lipids decay (e.g., in the 120–140 ppm and 20–40 ppm ranges), whereas signals from other components increase (e.g., in the 165–185 ppm and 40–70 ppm ranges), which is consistent with the transfer of ^1^H magnetization from the cell membrane to extramembraneous components. The calculated build-up distance of 2–3 nm from Fig. 5 aligns well with the approximate half-width of the lipid membrane (ca. 2.5 nm, Fig. 6c), reinforcing the validity of the distances expected from our spin diffusion model. For longer spin diffusion times (>100 ms), all NMR signals decay due the phase cycling that removes magnetization that *T*_1_(^1^H)-relaxed during the diffusion time^25^. The exponential build-up time constants *T*_BU_ associated with each spectral frequency (Fig. S6) provide a way to further characterize lengthscale of spin diffusion and distance from cell membranes.

**Fig. 7:**
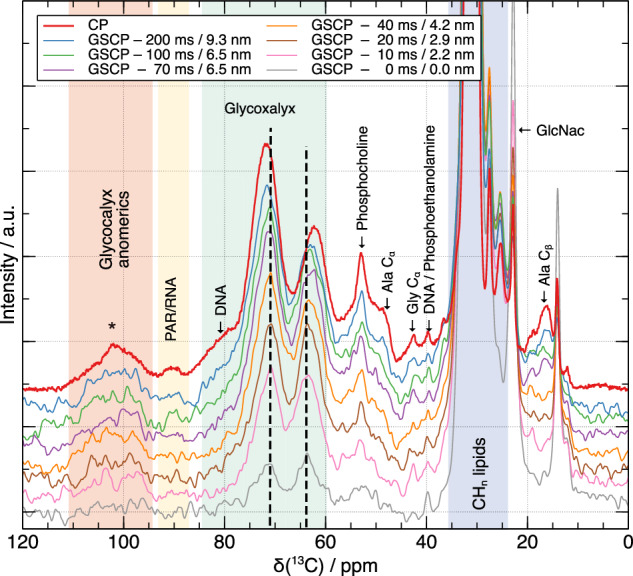
Interface-edited GSCP spectra at different spin diffusion times compared to CP spectra representing the spectrum for the bulk sample. Here, the distances reported correspond to the distance from cell membranes to where 80% of magnetization is found (Fig. 5). Dotted lines indicate glycerol CH (~63 ppm) and CH_2_ (~72 ppm) signals from phospholipid headgroups in the outer part of the cell membranes. * labels the position of a carbonyl spinning sideband.

Figure 7 shows the alkyl region of the interface-edited ^13^C GSCP spectra as a function of ^1^H spin diffusion time compared to a control ^13^C CP spectrum representing the spectrum for the whole sample. From the foregoing analysis, we expect the ^13^C spectrum at the shortest spin diffusion time of 10 ms to contain significant signal intensity only from ^13^C within 1 nm of the phospholipid acyl chain-headgroup junction, which are the phospholipid headgroups and the acyl chains themselves. This spin diffusion distance scale is confirmed by the intense signals at 71.4, 63.0 ppm in the ^13^C spectrum for 10 ms ^1^H spin diffusion from glycerol moieties in the phospholipid headgroups and little spectral intensity elsewhere except for the acyl chain spectral region (>33 ppm). Also present at this shortest ^1^H spin diffusion time are a sharp signal at 40 ppm from phosphoethanolamine ^13^C-NH_3_^+^ and a broad signal centered at ~53 ppm from phosphocholine ^13^CH_3_-N and -^13^CH_2_-N carbons. Phosphocholine and phosphoethanol amine are the most abundant phospholipid headgroups in mammalian cell membranes and are most likely represented in the GSCP ^13^C spectra at low spin diffusion times because the ^1^H in the highly mobile CH_3_ (phosphocholine) and NH_3_^+^ (phosphoethanolamine) have long T_1ρ_ times and so their magnetization is not filtered out by the T_1ρ_ filter in the GSCP experiments. As the spin diffusion time increases, the dominant change is the increase in intensity of ^13^C signals in the 60–85 ppm range, such that, for instance, the peak maximum in the secondary alcohol region increases from 71.4 ppm to ~73 ppm consistent with signals from C_3, 4_ carbons in glycocalyx sugar rings and that at 63 ppm moves to 61.9 ppm, consistent with the CH_2_OH of sugars in the glycocalyx. In contrast, there is an almost complete absence of signal intensity in the GCSP spectra in the 87–93 ppm range where signals from RNA and poly(ADP ribose) (PAR) are expected (PAR synthesis occurs as a result of cell death as part of sample preparation). RNA and PAR anomeric carbons are the only abundant carbons expected to give ^13^C signals in this chemical shift range, and both RNA and PAR are intracellular sugars; the absence of signals in this range in the GSCP spectra therefore confirm that the GSCP spectra are efficiently filtered for extramembraneous signals and exclude intracellular signals.

As spin diffusion times are increased and the magnetization is distributed over greater distances, interface-edited GSCP spectra gradually become more similar to bulk CP spectra to the point that the 200 ms spin diffusion time closely matches that of the CP spectrum, suggesting that a considerable contribution to the CP spectrum in the sugar region comes from the glycocalyx, but the GSCP spectrum even at this long spin diffusion time still does not show the characteristic signals from DNA or RNA, confirming the spectral editing effect.

### Multi-dimensional GSCP experiments to increase information content of the molecular fingerprint: GS-PDSD

**Fig. 8:**
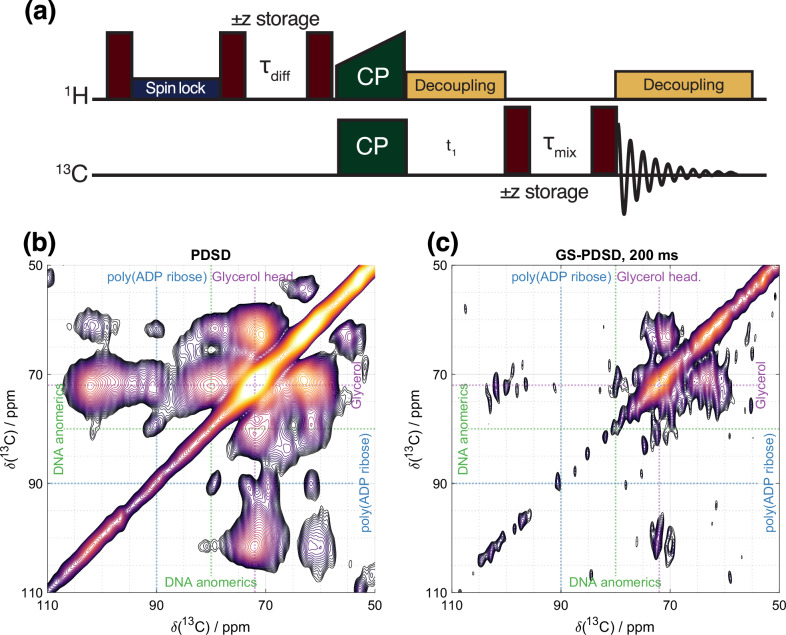
The application of 2D GS-PDSD experiments. **a** pulse sequence of a 2D ^13^C-^13^C interface-edited GS-PDSD experiment **b** ^13^C-^13^C PDSD correlation spectrum compared to **c** an interface-edited ^13^C-^13^C GS-PDSD correlation spectrum at 200 ms spin diffusion time/50 ms PDSD mixing time. More spin diffusion times and wider spectra widths are plotted in Fig. S7.

To improve spectral resolution and information content, we next combined GS spatial editing with 2D ^13^C-^13^C homonuclear correlation experiments (Fig. 8). We used a proton-driven ^13 ^C spin diffusion step to generate the spectral correlations, preceded by a GSCP step to spatially encode the ^1^H magnetization close to cell interfaces (Fig. 6). This pulse sequence has two distinct spin diffusion times: (i) the GSCP diffusion time is relayed in a network of protons and driven by ^1^H-^1^H dipolar interactions, that probe distances from cell interfaces. (ii) the PDSD diffusion time is relayed by ^13^C-^13^C dipolar interactions, which is assisted by the ^13^C-^1^H dipolar coupling that broadens the lines and results in a better ^13 ^C resonances overlap. This proton-driven diffusion time encodes ^13^C-^13^C distances that are represented by off-diagonal peak intensity. ^13^C spin diffusion can be enhanced by applying ^1^H recoupling schemes^26^ during the mixing time, e.g., as in a DARR experiment^27^. These schemes are particularly advantageous at high fields, fast MAS rates, and for samples with sharp spectral features. Since these conditions were not met in our study, ^1^H recoupling was not employed.

Figure 8 shows GS-PDSD spectra of a ^13^C-glucose labeled VSMC sample. As for 1D GSCP spectra, the interface-edited GS-PDSD should be compared with a PDSD spectrum recorded in identical conditions, in this case with an identical PDSD mixing time and an identical number of time increments meaning that the resolution in the indirect dimension can also be directly compared. The characteristic ^13^C correlation signals from glycerol in the phospholipid headgroups of the cell membranes appear at 50 ms ^1^H spin diffusion time, which acts as a reference spin diffusion time for when significant ^1^H magnetization reaches the outer part of the cell membrane. At 200 ms spin diffusion, correlation signals from glycocalyx sugars appear (~70, 75 ppm and ~80, 75 ppm and ~100, 72–75 ppm). The bulk PDSD spectrum has clear signals from DNA and poly(ADP ribose), both nuclear sugars, (the latter produced by cells during cell death in tissue harvesting), which are not observed even at long spin diffusion times in the GSCP-edited PDSD spectra, again confirming the spectral editing by the GSCP filter. Thus, the 2D GS-PDSD correlation spectrum in Fig. 8 can be confirmed as a 2D correlation spectrum of the cell glycocalyx.

## Conclusion

We have advanced the application of the ^13^C Goldman-Shen experiments to generate spectral fingerprints of cell glycocalyx in intact mammalian cells in situ in their native extracellular matrix. This approach minimizes the need for extensive and potentially denaturing sample preparation, making it well-suited for probing cells in their native environments. This development has significant implications for structural biology as it allows us to explore cell interfaces in a dehydrated state that does not requires further preparation, maintaining a context that is still closely aligned with their natural environment.

Our work builds upon the refined Goldman-Shen-CP experiment, introducing a more precise interface-editing that has been extended to encompass interface-edited ^13^C-^13^C PDSD correlation spectra. By combining measurements of the proton spin diffusion coefficient with a simple spin diffusion model employing heat equations, we have determined that proton magnetization can extend approximately 10 nm from cell membranes. This length scale is particularly well-suited for NMR investigations of cell interfaces in general and the glycocalyx in particular. Overall, our work provides a valuable tool for comparing the glycocalyx across different cell lines, paving the way for future investigations.

## Materials and methods

Vascular Smooth Muscle (VSMCs) initially harvested from fresh adult bovine aortae were cultured in ^13^C enriched DMEM media using the methods previously developed in the group^28–30^, as described in more details in the Supplementary Methods. One day after confluency, cells were harvested by washing them with 10 mL of phosphate buffer saline (PBS, Dulbecco’s Phosphate-Buffered Saline, Gibco), then scrapping, pelleting scrapped cells by centrifugation (2 min, 3260 rcf) and discarding the supernatant. Cells were finally frozen in liquid nitrogen and freeze-dried.

NMR experiments at 600 MHz ^1^H Larmor frequency 14.1 T were performed on a Bruker standard bore instrument, which was equipped with an Avance Neo console and a 4 mm CPMAS double resonance iProbe operating at room temperature. Chemical shifts were referenced using a secondary resonance setting the glycine-Cα resonance at 43.1ppm. Experiments used 10 kHz MAS, and 100 kHz proton decoupling during the acquisition, 2 ms 60 kHz CP contact time, and 50 ms mixing time for the 2D PDSD spectra. 2D GS-PDSD spectra were recorded in about 2 days (128 time-increments × 1024 scans). The pulse sequences are available in the supplementary information.

Data were zero-filled, apodized using an appropriate exponential decay function (line broadening up to 50 Hz), Fourier-transformed, and phase-corrected in Topspin. The NMR data were subsequently analyzed with MATLAB 2022b (MathWorks) using data loading RBNMR scripts and home-written scripts in order to more conveniently analyze and visualize the data.

### Reporting summary

Further information on research design is available in the Nature Portfolio Reporting Summary linked to this article.

## Supplementary information

SI

Reporting Summary

## Data Availability

All raw experimental data files, supporting code and pulse sequences are available in the Cambridge Research Repository, Apollo, with the identifier: https://doi.org/10.17863/CAM.112385.
