# Supplementary material for: Interface-edited solid-state NMR to study cell interfaces
Source: Commun Chem. 2025 Mar 22;8:86. doi: 10.1038/s42004-025-01473-7 (PMC11929740; doi:10.1038/s42004-025-01473-7)
Supplement: Supplementary file 1 — SI [file 42004_2025_1473_MOESM1_ESM.pdf]

Supplementary Information:
Interface-edited solid-state NMR to study cell interfaces

Thomas Kress [1], Melinda J. Duer [1*]

[1] Yusuf Hamied Department of Chemistry, University of Cambridge, Cambridge, CB2 1EW, United Kingdom

A. *Supplementary Materials and methods: Cell culture*

The cells were grown in DMEM complete media which were prepared from low (1 g·L^{-1}) glucose Dulbecco's Modified Eagle Medium (DMEM, Invitrogen). DMEM was supplemented to 9% v/v Foetal Calf Serum (Invitrogen), 30µg·mL^{-1} L-ascorbic acid 2-phosphate sesquimagnes (Sigma), and 10mL·L^{-1} of L-glutamine-penicillin-streptomycin solution (Sigma Aldrich, L-glutamine 200 mM, penicillin 10,000 units, streptomycin 10 mg·mL^{-1}). ^{13}C glucose enriched media used no glucose DMEM media supplemented with the 5.5mM ^{13}C glucose (Cambridge isotopes) instead of low glucose DMEM and supplemented as before. The cells were incubated at 37°C in humidified air containing 5% CO_2, and DMEM complete media were renewed every 3 days.

Before confluency, cells were detached with 10 mL/T175 of 0.025% trypsin/EDTA solution left for 5 minutes at room temperature. The digestion of adhesion proteins by trypsin was quenched by adding an equal volume of media. After washing the surface with the solution to detach the cells, solutions were filtered using a 40 µm cell strainer to remove cell clumps, and pelleted at 270 rcf for 3 minutes. Cells were resuspended in ca. 1 mL of ^{13}C glucose enriched media and split between the number of culture dishes required to pack a full 4 mm rotor, which was 10x145 mm Petri dishes (Cellstar) when the cells were harvested one day after confluence.

Harvesting happened the day following cell confluence and consisted in washing cells with 10 mL of phosphate buffer saline (PBS, Dulbecco's Phosphate-Buffered Saline, Gibco, before scrapping, pelleting by centrifugation (2 min, 3260 rcf) and discarding the supernatant. Cells were frozen in liquid nitrogen and freeze-dried in a Virtis Wizard 2.0 freeze dryer in a < 40 Pa vacuum.

A. ^{1}H-^{13}C INEPT

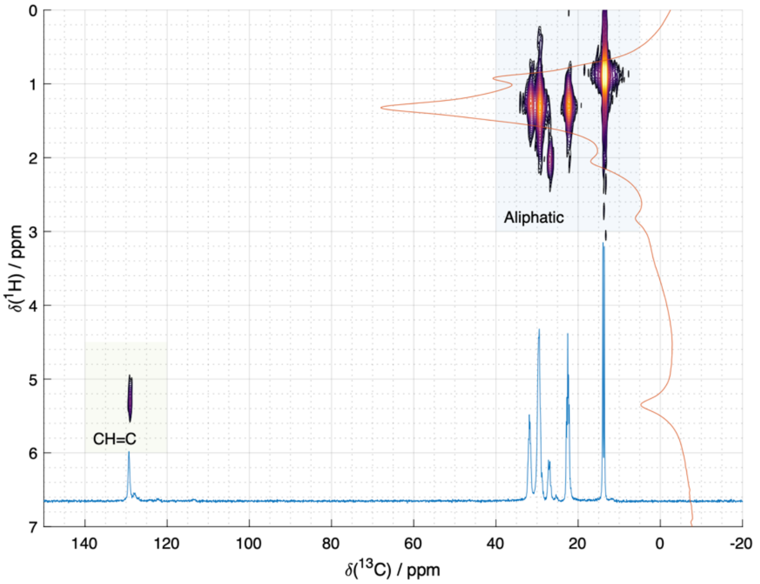

Figure S1 2D MAS 1H-13C INEPT-based heteronuclear shift correlation ssNMR spectrum of a VSMC cell sample labelled with U-13C Glucose. The 1H and 1H-13C INEPT 1D spectra are also displayed.

B. Selection of lipid magnetization is cleaner with a $T_{1\rho}(^1H)$ filter

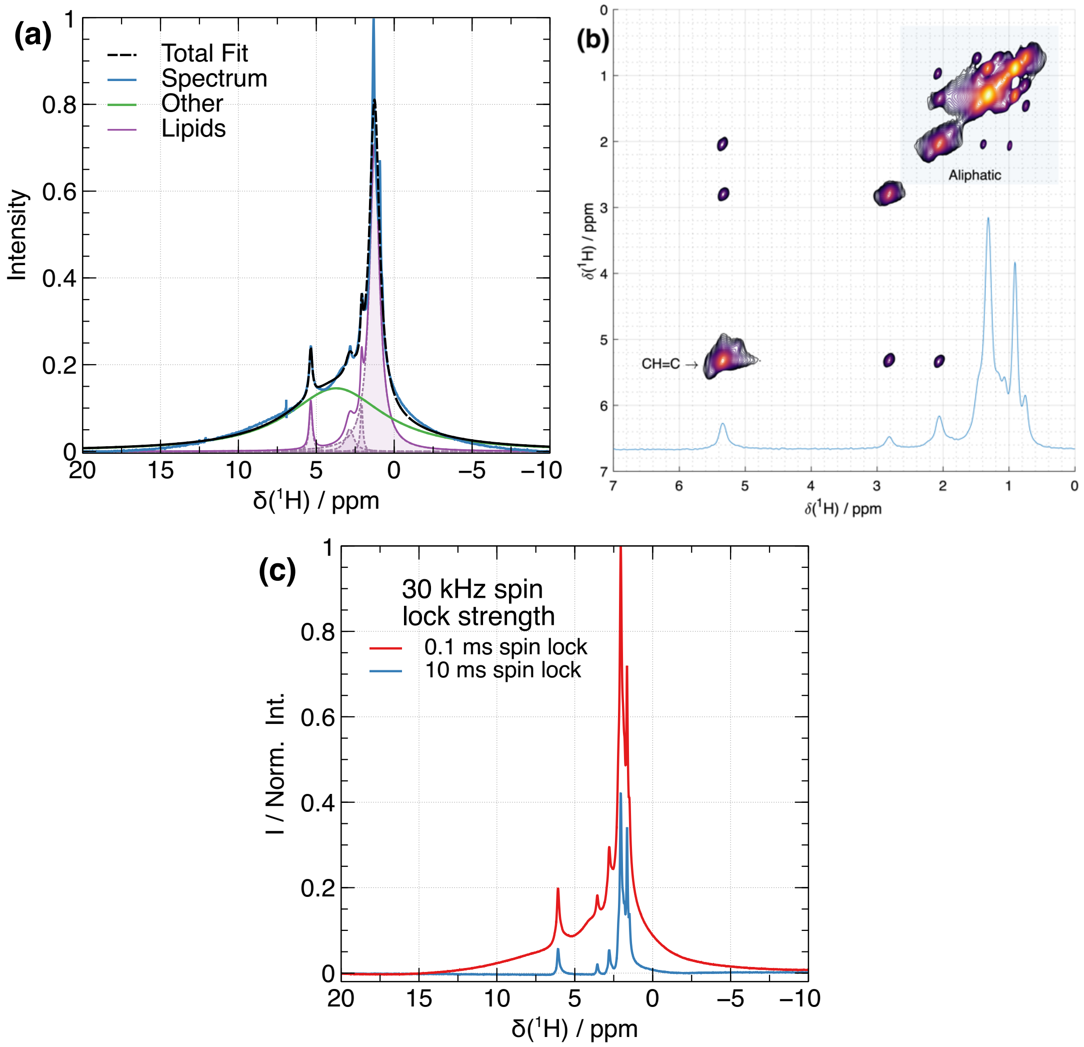

Figure S2 (A) The ^{1}H NMR spectrum of a VSMC cell sample labeled with U-^{13}C glucose reveals a superposition of sharp lipid signals (long T_2) over a broad background signal attributed to proteins (short T_2). The spectrum was fitted with a broad component representing extramembranous elements and sharp components (purple) corresponding to lipids. These fits qualitatively illustrate the presence of sharp lipid peaks superimposed on a broad, inhomogeneously broadened signal from other cellular components (green). (B) 2D TOCSY MAS 1H-1H correlation spectrum(10 ms at 30 kHz spin lock, 10 kHz MAS) showing 1H through space correlations. All the peaks are connected with a network of cross-peaks, suggesting that a single molecule is responsible for the sharp peaks. (C) ^{1}H NMR spectrum of a VSMC cell sample labelled with U-^{13}C glucose recorded after a 90º pulse and a 30 kHz spin lock

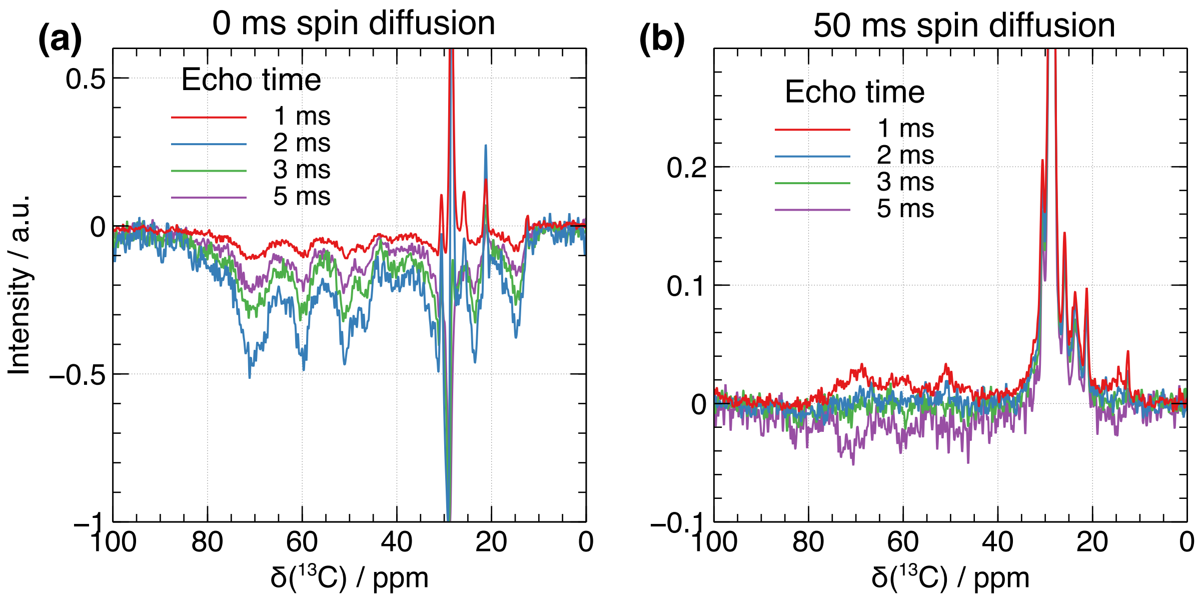

Figure S3 Effect of an increasing T_2(^{1}H) filter time in the Goldman-Shen experiment using a Hahn echo as a relaxation filter to select lipid proton magnetization. The spin diffusion time was set to (a) 0 ms: Despite a very long T_2(^{1}H), protein ^{1}H signals are never fully suppressed; (b) 50 ms spin diffusion GSCP spectra showing the sign inversion of protein signals.

C. <u>*$T_{1\rho}$(^{1}H) relaxation and contrast as a function of spin lock strength*</u>

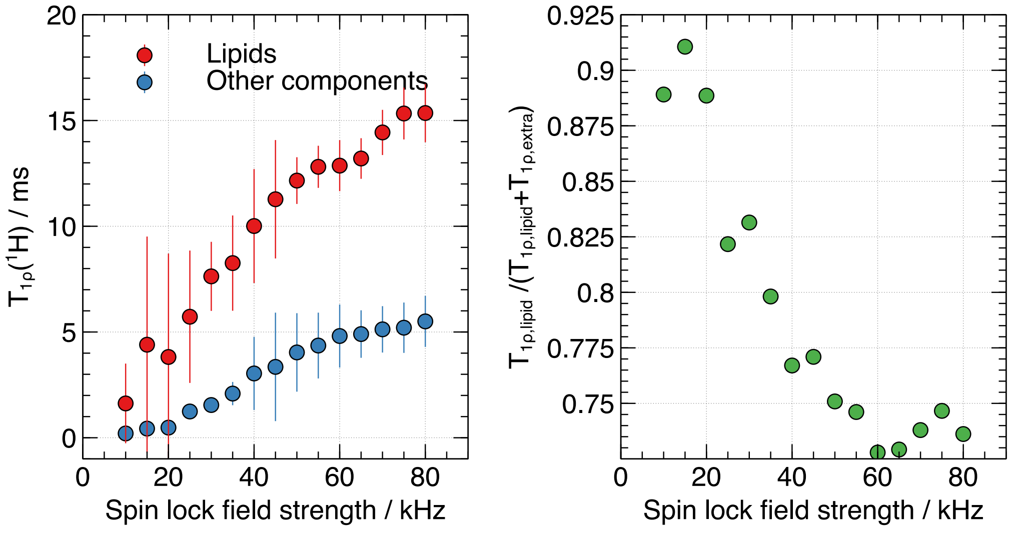

Figure S4 $T_{1\rho}$ relaxation times and contrast as a function of the spin lock field strength, and corresponding

D. *Measurement of spin diffusion coefficient.*

Although proton spin diffusion coefficient were initially calibrated on diblock copolymers by measuring the magnetization transfer rates between blocks with Goldman-Shen experiments[1,2], the measurement of spin diffusion coefficients was mainly made possible by Chen and Schmidt-Rohr, and later Walder et al, who developed pulse sequences based on REDOR filters [3,4].

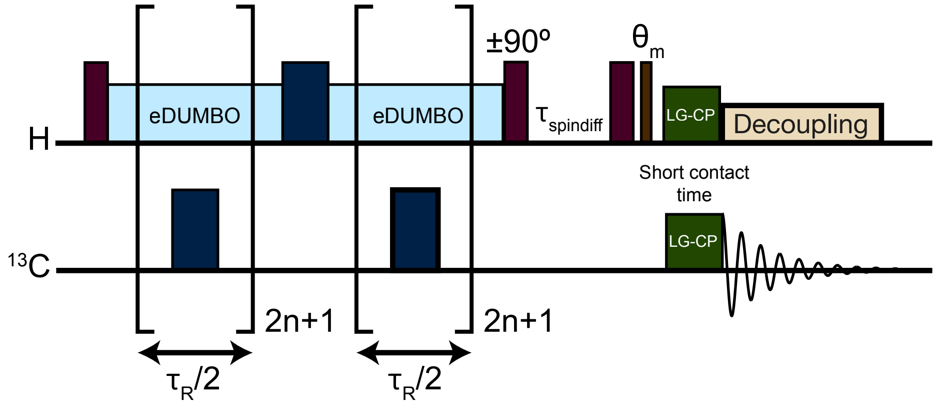

Figure S5 A hole-burn sequence to measure ^{1}H spin diffusion coefficients

In brief, the NMR pulse sequence to measure proton spin diffusion coefficients can be divided into three steps:

1. A hole burning step that creates a "hole" of magnetization of known radius around an observable X nucleus (e.g. ^{13}C) using a rotational echo double resonance (REDOR) filter
2. A hole filling step of variable duration during which ^{1}H magnetization flows into the "hole" of magnetization.
3. A CP detection step that follows the recovery ^{1}H magnetization in the hole of magnetization

Solving the diffusion equation (equation 1) using the spherical Laplace operator, neglecting T_1 relaxation, and using a perfect sphere as the initial conditions is [4]:

$$M(0,t) = M_\infty \left[1 - erf\left(\frac{r_{1/2}}{\sqrt{4Dt_{diff}}}\right) + \frac{r_{1/2}}{\sqrt{\pi D t_{diff}}}\, exp\left(\frac{{r_{1/2}}^2}{4Dt_{diff}}\right)\right]$$

Where D is the diffusion coefficient, t_{diff} is the diffusion time during which the hole is being filled, and $r_{1/2} = \sqrt[3]{0.8\, \frac{\mu_0}{4\pi} \hbar \gamma_H \gamma_X f_{scal}\, t_{deph}}$ is the heteronuclear distance at which half the magnetization is dephased [3], with t_{deph} being the dephasing time, f_{scal} being the scaling factor accounting for the attenuation of the heteronuclear dipolar interactions by homonuclear decoupling, γ_H and γ_X being the gyromagnetic ratios, and μ_0 being the vacuum permeability.

The measured value of the proton spin diffusion coefficient in polystyrene (0.33 ± 0.08 $nm^2.ms^{-1}$) matched the value found in the literature, and was similar to the value found in a cell sample (0.28 ± 0.07 $nm^2.ms^{-1}$).

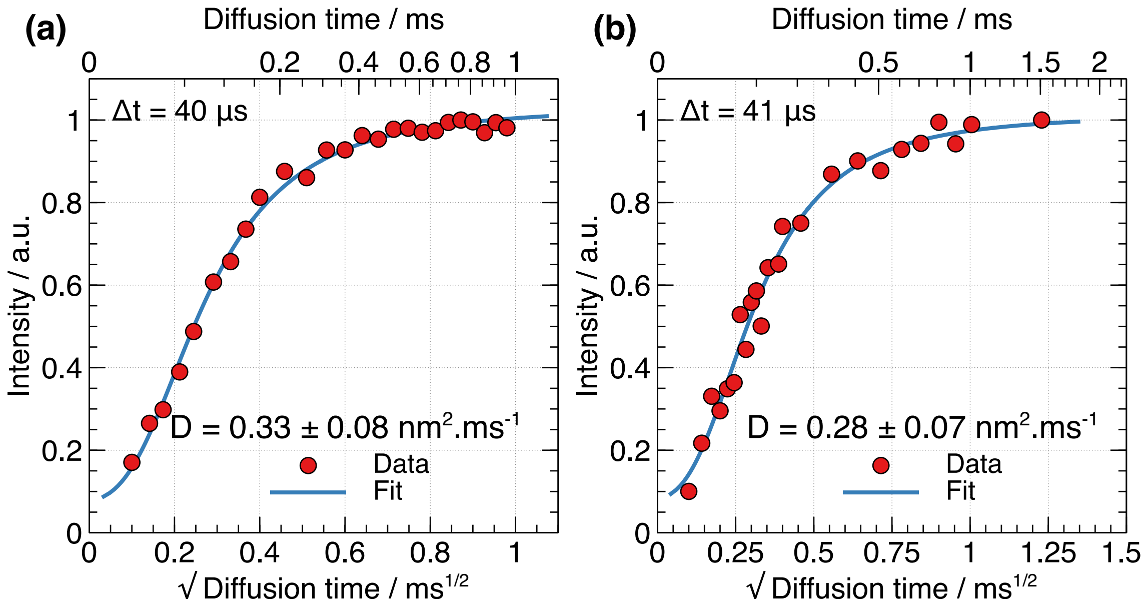

Figure S6 Measurement of ^{1}H spin diffusion coefficients using a hole burning — hole filling experiment. (a) Polystyrene; (b) Natural abundance VSMC sample prepared in the same conditions as the interface-edited experiments. Recorded at 10kHz / 400MHz.

E. Diffusion build-up plot

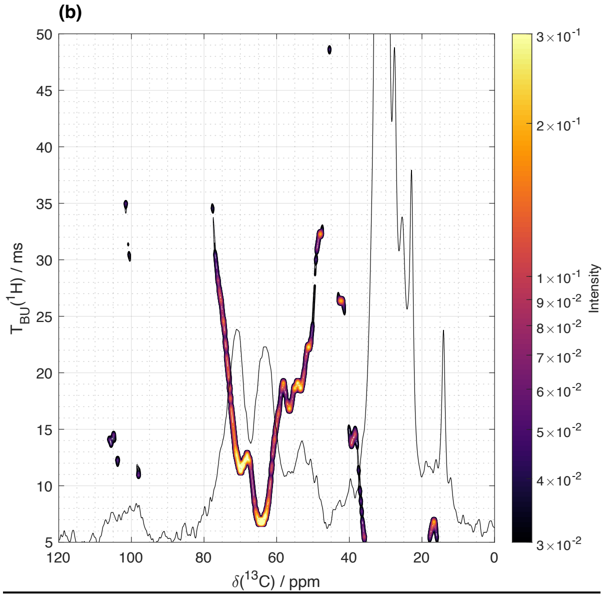

Figure S6 Exponential build-up time constants T_{BU} (defined as $I(t_{diff}) = I_0\ exp(-t_{diff}/T_{BU}) \times\ (1 - exp(-t_{diff}/T_1))$) plotted against the ^{13}C chemical shift. The colormap represent the intensity I_0. Further characterization of the glycocalyx can be made in this GSCP approach by quantifying the exponential build-up time constants. Signals in corresponding to build-up times of 7-12 ms correspond to phospholipid headgroups and those with significantly longer build-up times (20 – 30 ms) to carbons in the glycocalyx.

F. Multi-dimensional GSCP experiments to increase information content of the molecular fingerprint: GS-PDSD

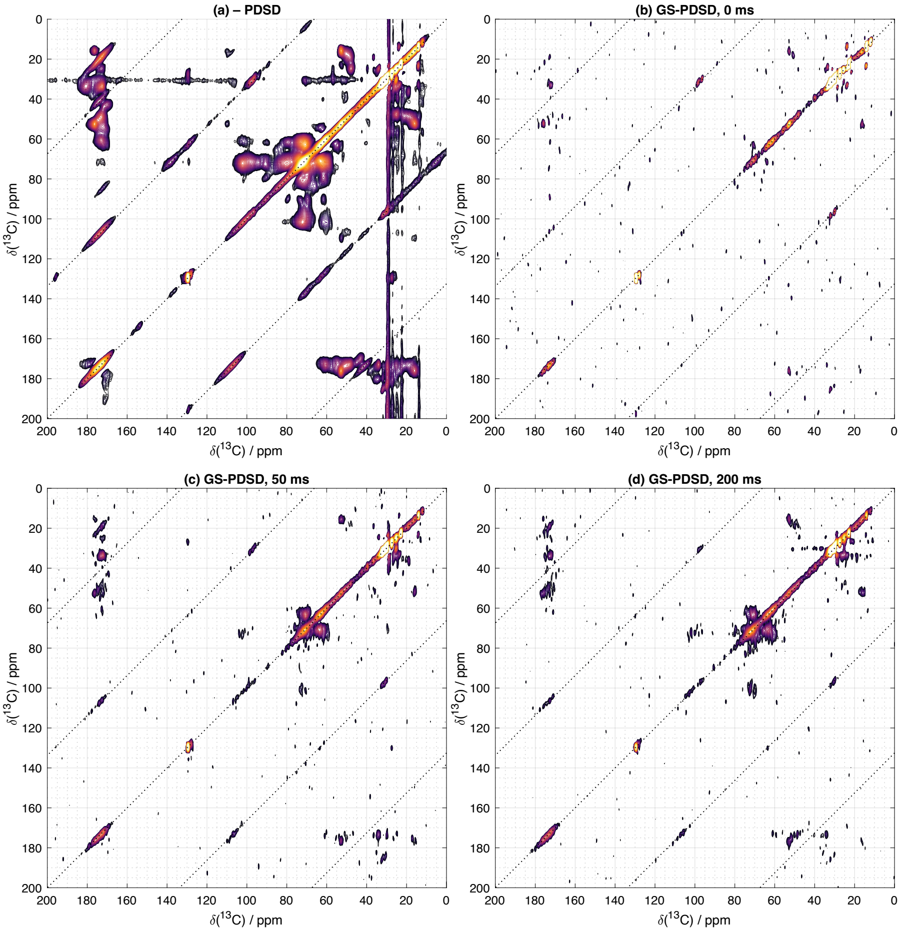

Figure S7 ^{13}C-^{13}C PDSD correlation spectrum compared to an interface-edited ^{13}C-^{13}C GS-PDSD correlation spectrum at 200ms spin diffusion time / 50 ms PDSD mixing time. Spinning sidebands are indicated with dashed lines.

G. <u>Pulse sequence: Spin-lock GSCP:</u>

As a pseudo-2d experiment

```
; slgscp_pz.tk506 (TopSpin 4.1.4)
; spinlock Goldman-Shen experiment - Storage + Z - CP 1H -> 13C
; Written by tk506 09/06/22

;parameters:
;ns : 8 * n
;d1 : recycle delay

; ### T1rho filter ###
;p3 : proton 90 at power level pl3
;p6 : spin lock time
;pl6: spin lock power level
;cnst6: spin lock strength /kHz

; ### Spin diffusion ###
;d8 : spin diffusion delay

; ### For CP ###
;p15 : contact time at pl1 (f1) and pl2 (f2)
;pl1 : X power level during contact
;pl2 : 0w not used
;pl12 : pulse and decoupling power level (if not pl13)
;d1 : recycle delay
;p31 : spinal64 pulse length
;cpdprg2 :spinal64
;spnam0 :ramp70100.100

;$COMMENT=GSCP
;$CLASS=Solids
;$DIM=1D
;$TYPE=direct excitation
;$SUBTYPE=simple 1D
;$OWNER=tk506

prosol relations=<solids_cp>

define list<delay> vd_list = <$VDLIST>

#include <trigg.incl>
                      ;10 usec trigger pulse at TCU connector I cable 6

"cnst53 = (1/(4*p3))*1000000"
"plw6=plw12*pow(10,(2*log10(cnst6/cnst53)))"
"d6 = 1/cnst6"

1 ze
2 d1 do:f2            ;recycle delay, decoupler off in go-loop
#include <p15_prot.incl>
                      ;make sure p15 does not exceed 10 msec
                      ;let supervisor change this pulseprogram if
                      ;more is needed
#ifndef lacq   /* disable protection file for long acquisition change decoupling power
!!! or you risk probe damage */
               /* if you set the label lacq (ZGOPTNS -Dlacq), the protection is
disabled */

#include <aq_prot.incl>
                      ;allows max. 50 msec acquisition time, supervisor
                      ;may change  to max. 1s at less than 5 % duty cycle
                      ;and reduced decoupling field
#endif

  1u fq=cnst21:f2
  10u pl12:f2 pl1:f1  ;preselect pl12 drive power for F2, pl1 for F1
  trigg               ;trigger for scope, 10 usec

       ; ### Spin locking protons
  (p3 ph1):f2 ; proton 90 deg
  (p6 pl6 ph2):f2     ;proton spin lock pulse at pl14
```

```
        ; ### +z storage for spin diffusion
        (p3 pl12 ph3):f2
  vd_list
  ; ### Cross polarisation with usual phase cycling
  trigg
  p3:f2 ph11          ;proton 90
  0.3u
  (p15 pl1 ph12):f1 (p15:sp0 ph10):f2        ;contact pulse with square or ramp

  1u cpds2:f2         ;pl12 is used here with tppm, pl13 with cwlg, cwlgs
  go=2 ph31
  1m do:f2            ;decoupler off
  1m
  10m mc #0 to 2 F1QF(vd_list.inc)
HaltAcqu, 1m          ;jump address for protection files
exit                  ;quit


ph0=  0
ph1=  0 1 2 3
ph2=  1 2 3 0
ph3=  2 3 0 1  2 3 0 1  0 1 2 3  0 1 2 3
; ++++++++++++++++++ ----------------
ph10= 0
ph11= 3 1 3 1  3 1 3 1  1 3 1 3  1 3 1 3
ph12= 0 0 2 2  1 1 3 3
ph31= 0 2 2 0  1 3 3 1
```

H. Pulse sequence: GS-PDSD:

```
; GS-PDSD
;avance-version (20/11/23)
;
;version: 1.0/ TS3 / 6/24/2011
;
;basic 2D homonuclear exchange experiment
;written by HF and JOS
;updated JOS 6/24/2011
;checked by JOS 24/6/2011
;
;General Comment
;2D exchange NMR in rotating solids using PDSD mixing (PL14=120db)
;or DARR/RAD mixing with pl14 set such that the RF field is n*spin rate, n=1-3
;rotor synchronized, set cnst31=spin rate in Hz
;rotor synchronize in F1 choose n*spin rate for SWH in F1
;DARR Dipolar assisted rotational resonance (K. Takegoshi et al Chem. Phys. Lett. 2001,
344, 631.
;RAD (RF assisted diffusion) C.R. Morcombe et al., JACS 2004, 126, 7196.
;
;requires modification to run under TS2.1 inquire with applications
;
;Avance III version
;parameters:
;d1 : recycle delay
;p1 : X 90 degree pulse
;p3 : H 90 degree pulse
;p15 : contact time at plw1 (rf-channel f1) and spw0 (rf-channel f2)
;pl11 : rf-power on X for 90 degree pulses
;pl12 : rf-power for 1H pi/2 pulse p3 and standard proton decoupling
;pl13 : e.g. used in tppm13
;pl14 : rf-power for RAD-DARR mixing pulse, calculate or measure B1 field = 1 * rotation
rate
;cnst31 : spinning rate in Hz
;d8 : mixing time,recalculated for multiple rotor period, max. 500 msec
;sp0 : rf-power for contact pulse of 1H channel (rf-channel f2)
;spnam0 : file name for shaped CP pulse
;cpdprg2 : 1H decoupling sequence (spinal64, tppm15, cw, etc.)
;FnMODE: TPPI, States-TPPI or STATES
;ns : 16*n
;SWH[F1]: integer multiple of rotor frequency
;zgoptns: -Dlacq, -Dlcp15, or blank
```

```
;
;
; ### T1rho filter ###
;p3 : proton 90 at power level pl3
;p6 : spin lock time
;pl6: spin lock power level
;cnst6: spin lock strength /kHz
;d9; GSCP spin diffusion time

;$CLASS=BioSolids
;$DIM=2D
;$TYPE=cross polarisation
;$SUBTYPE=homonuclear correlation
;$COMMENT=exchange NMR in rotating solids using RAD/DARR for improved spin exchange, rotor
synchronised


prosol relations=<solids_cp>


#include <Avance.incl>
#include <trigg.incl>


"cnst59=plw14"
"d31=1s/cnst31"

define pulse mixing
define delay darrmix
define loopcounter count
define loopcounter darr
"darr=d8/d31"                                                    /*setup for rotor
synchronized mixing time */
"mixing=(darr*d31)"
"l0=0"                                                                          /* house
keeping for detection in t1, start at t1=0 for flat baseline */
"in0=inf1"
"d0=1u"
"acqt0=-p1/2"
"darrmix=mixing"

"cnst53 = (1/(4*p3))*1000000"
"plw6=plw12*pow(10,(2*log10(cnst6/cnst53)))"
"d6 = 1/cnst6"

1 ze
#include <rot_prot.incl>
#ifndef lcp15
#include <p15_prot.incl>
                   ;make sure p15 does not exceed 10 msec
                   ;let supervisor change this pulseprogram if
                   ;more is needed
#endif
#ifndef lacq
                   ;disable protection file for long acquisition change decoupling power
!!! or you risk probe damage
                   ;if you set the label lacq (ZGOPTNS -Dlacq), the protection is disabled

#include <aq_prot.incl>
                   ;allows max. 50 msec acquisition time, supervisor
                   ;may change  to max. 1s at less than 5 % duty cycle
                   ;and reduced decoupling field
#endif
#include <darrmix2_prot.incl>    /*allows max. 500 msec mixing time for PDSD, 100m for
DARR*/
  darrmix
  d31
2 10m do:f2
  d1
  1u fq=cnst21:f2
  10u pl12:f2 pl1:f1    ;preselect pl2 drive power for F2
  trigg

    ; ### Spin locking protons
  (p3 ph20):f2 ; proton 90 deg
  (p6 pl6 ph21):f2 ;proton spin lock pulse at pl14
```

```
    ; ### +z storage for spin diffusion
    (p3 pl12 ph22):f2
  d9
  ; ### Cross polarisation with usual phase cycling
  trigg
  p3:f2 ph23              ;proton 90
  0.3u


  (p15 ph2):f1 (p15:sp0 ph10):f2        ;contact pulse
 if "l0==1"{
   "d0=in0"
    }
if "l0>0"
  {                ;use cpdprg2=tppm15, SPINAL64 or XiX
  d0 cpds2:f2
}
  (p1 pl11 ph3):f1 (1u do):f2
  (mixing pl14 ph11):f2
  (p1 ph5):f1 (1u cpds2):f2
  go=2 ph31
  1m do:f2
  10m mc #0 to 2 F1PH(ip2,id0 & iu0)

HaltAcqu, 1m
6 exit

ph20 = 1 3
ph21 = 0 2
ph22 = {3 1}*16 {1 3}*16
; + + - -
ph23 = {1 3}*16 {3 1}*16

;ph1=1 3
ph2=1
ph3={0}*8 {2}*8
ph5=0 0 2 2 1 1 3 3
ph10=0
ph11=0
ph31=0 2 2 0 1 3 3 1
     2 0 0 2 3 1 1 3


;$Id:$
```


1. Cheung, T. T. P. & Gerstein, B. C. 1H nuclear magnetic resonance studies of domain structures in polymers. *Journal of Applied Physics* **52**, 5517–5528 (1981).
2. Clauss, J., Schmidt-Rohr, K. & Spiess, H. W. Determination of domain sizes in heterogeneous polymers by solid-state NMR. *Acta Polym.* **44**, 1–17 (1993).
3. Chen, Q. & Schmidt-Rohr, K. Measurement of the local 1H spin-diffusion coefficient in polymers. *Solid State Nuclear Magnetic Resonance* **29**, 142–152 (2006).
4. Walder, B. J. *et al.* Measurement of Proton Spin Diffusivity in Hydrated Cementitious Solids. *The Journal of Physical Chemistry Letters* **10**, 5064–5069 (2019).
